# Prediction of Smoking Risk From Repeated Sampling of Environmental Images: Model Validation

**DOI:** 10.2196/27875

**Published:** 2021-11-01

**Authors:** Matthew M Engelhard, Joshua D'Arcy, Jason A Oliver, Rachel Kozink, F Joseph McClernon

**Affiliations:** 1 Department of Biostatistics & Bioinformatics Duke University School of Medicine Durham, NC United States

**Keywords:** smoking, smoking cessation, machine learning, computer vision, digital health, eHealth, behavior, CNN, neural network, artificial intelligence, AI, images, environment, ecological momentary assessment, mobile health, mHealth, mobile phone

## Abstract

**Background:**

Viewing their habitual smoking environments increases smokers’ craving and smoking behaviors in laboratory settings. A deep learning approach can differentiate between habitual smoking versus nonsmoking environments, suggesting that it may be possible to predict environment-associated smoking risk from continuously acquired images of smokers’ daily environments.

**Objective:**

In this study, we aim to predict environment-associated risk from continuously acquired images of smokers’ daily environments. We also aim to understand how model performance varies by location type, as reported by participants.

**Methods:**

Smokers from Durham, North Carolina and surrounding areas completed ecological momentary assessments both immediately after smoking and at randomly selected times throughout the day for 2 weeks. At each assessment, participants took a picture of their current environment and completed a questionnaire on smoking, craving, and the environmental setting. A convolutional neural network–based model was trained to predict smoking, craving, whether smoking was permitted in the current environment and whether the participant was outside based on images of participants’ daily environments, the time since their last cigarette, and baseline data on daily smoking habits. Prediction performance, quantified using the area under the receiver operating characteristic curve (AUC) and average precision (AP), was assessed for out-of-sample prediction as well as personalized models trained on images from days 1 to 10. The models were optimized for mobile devices and implemented as a smartphone app.

**Results:**

A total of 48 participants completed the study, and 8008 images were acquired. The personalized models were highly effective in predicting smoking risk (AUC=0.827; AP=0.882), craving (AUC=0.837; AP=0.798), whether smoking was permitted in the current environment (AUC=0.932; AP=0.981), and whether the participant was outside (AUC=0.977; AP=0.956). The out-of-sample models were also effective in predicting smoking risk (AUC=0.723; AP=0.785), whether smoking was permitted in the current environment (AUC=0.815; AP=0.937), and whether the participant was outside (AUC=0.949; AP=0.922); however, they were not effective in predicting craving (AUC=0.522; AP=0.427). Omitting image features reduced AUC by over 0.1 when predicting all outcomes except craving. Prediction of smoking was more effective for participants whose self-reported location type was more variable (Spearman *ρ*=0.48; *P*=.001).

**Conclusions:**

Images of daily environments can be used to effectively predict smoking risk. Model personalization, achieved by incorporating information about daily smoking habits and training on participant-specific images, further improves prediction performance. Environment-associated smoking risk can be assessed in real time on a mobile device and can be incorporated into device-based smoking cessation interventions.

## Introduction

### Background

Cigarette smoking is the leading cause of preventable deaths in the United States [1], and tobacco use is responsible for more than 7 million annual deaths worldwide [2]. Although most smokers are motivated to quit [3], fewer than 10% of quit attempts are successful [4], which has motivated ongoing efforts to develop more effective cessation strategies.

New, mobile device-based cessation interventions have improved 6-month [5] and 12-month [6] cessation outcomes, and success rates can be further improved [7] by tailoring interventions to individual users and momentary contexts, such as the user’s geographic location [8]. This paradigm has been formalized as the just-in-time adaptive intervention (JITAI) [9,10], wherein a cessation support system continuously monitors contextual factors through ecological momentary assessment (EMA; ie, repeated self-reporting), passive sensing, or a combination of the two, then provides context-sensitive support to smokers at times when it is most needed. By monitoring user physiology [11-14], geographic location [8,15], and recent smoking events [15], a cessation support system can estimate smoking risk from moment to moment and then intervene when the estimated risk is high.

However, most smoking cessation interventions neglect an important contributor to smoking behaviors—the smoker’s external environment. A growing body of evidence, collected in both laboratory and real-world settings, suggests that smoking risk is affected not only by internal factors but also by the smoker’s current environmental context. For example, images of personal smoking environments increase smoking behaviors and self-reported craving [16-19] and activate neural circuits associated with craving, contributing to subsequent smoking behaviors [20]. Consistent with these findings, a recent study showed that self-reported environmental conditions, such as being around other smokers or in a place where smoking is permitted, were stronger predictors of smoking lapse than internal states (eg, smoking urge) [21].

Current technologies can now quantify the effects of environmental factors on real-world smoking behaviors [22]. Wearable cameras and mobile devices allow images of daily environments to be acquired near continuously at low cost [23-26], and computer vision models, now highly accurate and optimized for mobile devices [27], can identify environmental features in these images. These features can then be linked to smoking and craving, along with other behaviors or outcomes of interest. This process is objective, does not require manual annotation of images, and scales to large data sets and study populations.

In a previous study, we demonstrated that computer vision could distinguish between daily environments where smokers commonly smoke and those where they rarely smoke. Using the approach outlined above, we also uncovered specific objects and settings associated with smoking versus nonsmoking environments [28]. These findings suggest that environmental features monitored via computer vision may provide important contextual information that can improve the prediction of momentary smoking risk. However, these two extremes, known smoking and nonsmoking environments, do not reflect the full range of environments that smokers encounter in their daily lives.

### Objective

In this study, we collected a representative sample of images of smokers’ daily environments through photograph-augmented EMA (photoEMA). In each assessment, participants self-reported recent smoking and their current craving level and then took a picture of their environment. A mobile-optimized convolutional neural network was trained to predict smoking risk and other outcomes relevant to smoking (craving, whether smoking was permitted in the current environment, and whether the participant was outside) based on environmental images and other participant-specific features. We hypothesized that out-of-sample prediction would be effective, providing a basis for an environment-aware JITAI, and that prediction performance could be improved through model personalization, in which images from a given participant are used to refine model predictions for that participant. We also aim to understand how model performance varies by location type, as reported by participants. Our final prediction model, QuitEye, was deployed on a mobile device and can assess environment-associated smoking risk and craving in real time to support environment-aware smoking cessation interventions.

## Methods

### Study and Participants

Recruitment and all study procedures were approved by the Duke University Health System Institutional Review Board, and written consent was obtained from all participants. Smokers (≥10 cigarettes per day for ≥2 years) aged ≥18 years were recruited from the Durham, North Carolina area. Participants were recruited from the community for a study of smoking behavior via printed and web advertisements and word-of-mouth. Participants were excluded if they regularly used noncigarette tobacco products (eg, e-cigarettes); currently used smoking cessation medications; planned to quit smoking, otherwise altered their smoking pattern, left the study area or anticipated a major life event during the study; had current or recent alcohol or drug abuse problems; or were pregnant, breastfeeding, or planning to become pregnant during the study. Eligible participants completed an initial visit to (1) biochemically verify their smoking status (ie, carbon monoxide breath test) and test for illicit drug use, (2) test for pregnancy, and (3) complete questionnaires on nicotine dependence and tobacco use history. Participants who met all eligibility requirements (n=52) then downloaded the photoEMA app (Metricwire) to their smartphone and were trained on its use. Following the 14-day photoEMA period, participants completed a follow-up visit during which an interview was conducted to assess drug and alcohol use, tobacco purchasing, and any other events that might have affected smoking (eg, illness) or daily living (eg, death in the family) patterns. Participants were compensated for up to US $350 in total, including daily (US $5) and weekly (US $50) incentives for high photoEMA completion. All procedures were observational, and no randomization or intervention was performed.

### PhotoEMA Collection

Participants completed the photoEMA assessments for 14 days.

#### Random Prompts

Participants specified their typical wakeful hours during the screening. They were prompted six times daily at randomly spaced intervals. The average interval between prompts was 120 minutes in duration. At each assessment, participants rated their current levels of urge to smoke (1 item) and affect and stress (11 items; not reported here). In addition, they captured a time-stamped image of their current location. Finally, they were prompted to label the location with a prepopulated list of common locations (eg, bedroom, office, car, or park), other location information (eg, indoors or outdoors and whether smoking was permitted), current activity (eg, working or running errands), social environment (eg, presence of others), and recent alcohol and caffeine use.

#### Smoking-Initiated Assessments

Participants were also instructed to complete assessments each time they smoked. They were asked how many cigarettes they smoked in this location on this occasion and all items from the random prompt assessments.

#### Across Prompt Types

Participants were instructed to delay responding if they were in situations or locations where responding to, or initiating prompts, would be distracting (eg, in a meeting) or dangerous (eg, while driving). Across assessments, participants were asked to compose pictures to avoid including other people but to otherwise leave environments as they are.

Craving data were dichotomized based on the median self-reported craving for all participants. Self-reported craving of a *moderate* or lower level was coded as negative, and self-reported craving of *quite a bit* or higher level was coded as positive. Other outcomes (smoking, whether smoking was permitted, and whether the participant was outside) were binary; therefore, no binarization was required.

### Convolutional Neural Network Model

QuitEye is based on MobileNetV2, a convolutional neural network architecture optimized for mobile devices [27] pretrained on ImageNet [29]. QuitEye also incorporates the following additional information: (1) participant age and sex, (2) known smoking locations inside and outside the home as indicated by self-report at baseline, (3) time since the last cigarette, (4) time of day and day of the week, and (5) a participant-specific indicator variable (in the longitudinal models only).

To determine the impact of each of these elements on prediction performance, we conducted ablation studies in which models *that did not incorporate* a given element were also trained and evaluated. For example, we trained a model *without* age and sex to assess the impact of these factors on prediction performance, and to estimate performance for a possible deployment in which these were not available. Models that did not incorporate image features were also trained and evaluated to determine the impact of the images on prediction performance.

**Figure 1:**
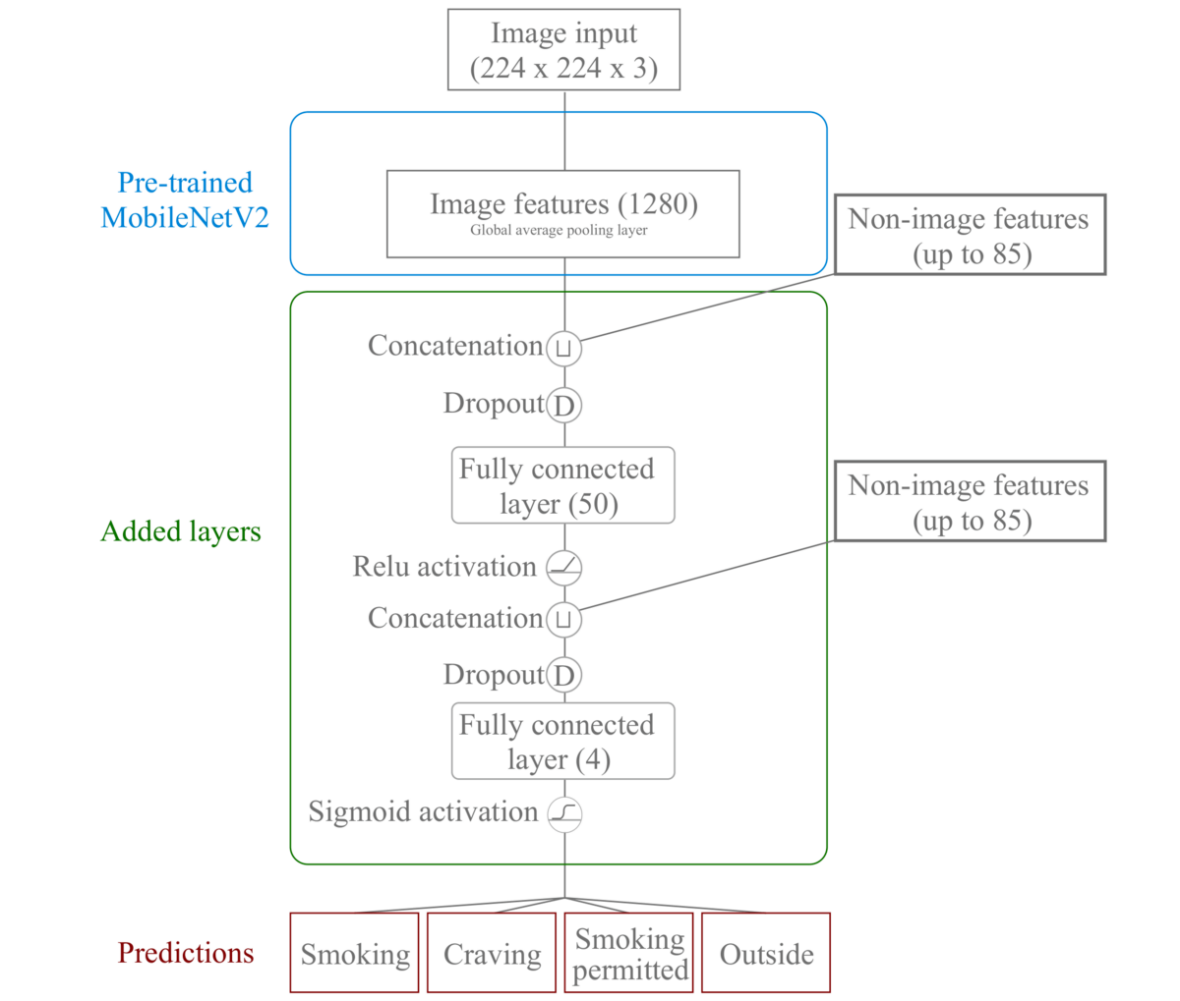
Diagram of QuitEye, which extracts image features using the MobileNetV2 convolutional neural network, then predicts smoking status, craving, whether smoking is permitted, and whether the participant is outside based on a combination of image features and additional data collected from participants with a mobile device.

QuitEye is a multi-task architecture that jointly predicts four binary outcomes: smoking, craving, whether smoking is permitted, and whether the participant is outside. Prediction of whether the participant is outside was included both to contextualize other performance figures and because inside or outside status is associated with smoking behaviors. Nonimage features were concatenated with image features from the global pool layer of MobileNetV2, and a single hidden neural network layer (rectified linear unit activation) was applied. Nonimage features were again concatenated to the output of this layer, and a second fully connected layer (sigmoid activation) was then used to predict each of the four binary outcomes. The QuitEye architecture is shown in Figure 1.

### Training and Evaluation

QuitEye was trained using Tensorflow v1.15 in Python v3.7 on a single Titan XP GPU. MobileNetV2 parameters were initialized to values learned on ImageNet [29], and all parameters were fine-tuned. Two training and evaluation procedures were used to evaluate out-of-sample performance (ie, *out-of-the-box* performance, without first learning from images from a given participant) and personalized model performance (ie*,* after learning from a subset of that participant’s images).

Out-of-sample performance was assessed by training and evaluating the model using nested cross-validation [30] with five outer folds and five inner folds. In the nested cross-validation procedure, out-of-sample performance was evaluated on each outer fold after developing the model using data from the remaining folds. In each development set, an inner cross-validation procedure was used to determine the optimal hyperparameter settings.

Personalized model performance was assessed by developing the model with data from all participants from days 1 to 10, then evaluating it on data from days 11 to 14. Images used in model development were divided at random into training (80%) and validation (20%) sets.

Hyperparameters included the width of the hidden layer (Figure 1), hidden layer dropout rate, and learning rate. Performance was evaluated using the area under the receiver operating characteristic curve (AUC) for each of the four prediction tasks.

Additional models were trained using the procedures outlined above to quantify the impact of additional (nonimage) features on performance. Features were categorized as (1) baseline information, including participant demographics and smoking habits; (2) information that could be collected via mobile devices, including the time elapsed since the participant last smoked and the time of day; and (3) a unique participant identifier, which was incorporated as a categorical feature in the personalized models only. Including this identifier adds participant-specific parameters to the model, allowing predictions to be explicitly personalized. However, even when this identifier is omitted, the personalized model development scheme (ie, training on days 1-10 from all participants) allows the model to learn from each participant’s previously visited locations when predicting their current risk.

### Mobile Device Implementation (QuitEye)

A nonpersonalized (out-of-sample) model incorporating image features only was implemented in TensorFlow Lite to allow prediction via mobile devices. Other features were omitted so that predictions could be made based on images only without additional data collection. A prototype mobile app was built using Flutter or Dart and tested on Google Pixel 3 (Android). QuitEye is applied to individual frames from a live video feed at a rate of approximately eight samples per second and is configured to display smoking and craving predictions corresponding to each frame.

### Data Availability

The data sets analyzed in this study are not publicly available because they contain images of participants’ personal daily environments that cannot be deidentified. However, the code supporting this work is available from the corresponding author upon reasonable request.

## Results

### Demographics and Descriptive Statistics

Of the 77 individuals screened for the study, 52 (68%) were eligible and consented to participate. Four participants were withdrawn or lost to follow-up, and the remaining 48 participants completed the study. One participant completed their study visits remotely because of in-person visit restrictions related to COVID-19. Among the participants who completed the study, a total of 8008 images were collected, 3648 (45.55%) of which were from completed random prompts and 4360 (54.45%) of which were from completed smoking prompts. Demographic characteristics, image details, and other descriptive statistics are presented in Table 1.

**Table 1:** Demographics and descriptive statistics (N=48).

Characteristics				Values
**Demographics**				
	**Sex**			
		Female:male	32:16	
		Female, n (%)	32 (67)	
	**Age (years)**			
		Value, median (IQR)	40.5 (31-49)	
		Value, range	19-64	
	**Race, n (%)**			
		White	31 (65)	
		Black or African American	19 (40)	
		American Indian	1 (2)	
		Native Hawaiian or Pacific Islander	1 (2)	
	**Ethnicity, n (%)**			
		Not Hispanic or Latino	46 (96)	
		Hispanic or Latino	2 (4)	
**Smoking history**				
	**Cigarettes per day (weekday)**			
		Value, median (IQR)	15 (12-20)	
		Value, range	7-30	
	**Cigarettes per day (weekend)**			
		Value, median (IQR)	15 (14-20)	
		Value, range	10-30	
	**Fagerstrom test of nicotine dependence**			
		Value, median (IQR)	6 (4-7)	
		Value, range	2-9	
**Images**				
	**Total images taken**			
		Value, median (IQR)	163 (117-200)	
		Value, range	63-406	
	**Images when smoking**			
		Value, median (IQR)	87 (67-132)	
		Value, range	25-322	
	**Images when craving**			
		Value, median (IQR)	58 (19-99)	
		Value, range	1-210	
	**Images when smoking permitted**			
		Value, median (IQR)	122 (96-160)	
		Value, range	25-388	
	**Images when outside**			
		Value, median (IQR)	45 (20-78)	
		Value, range	3-183	

### Model Performance

**Figure 2:**
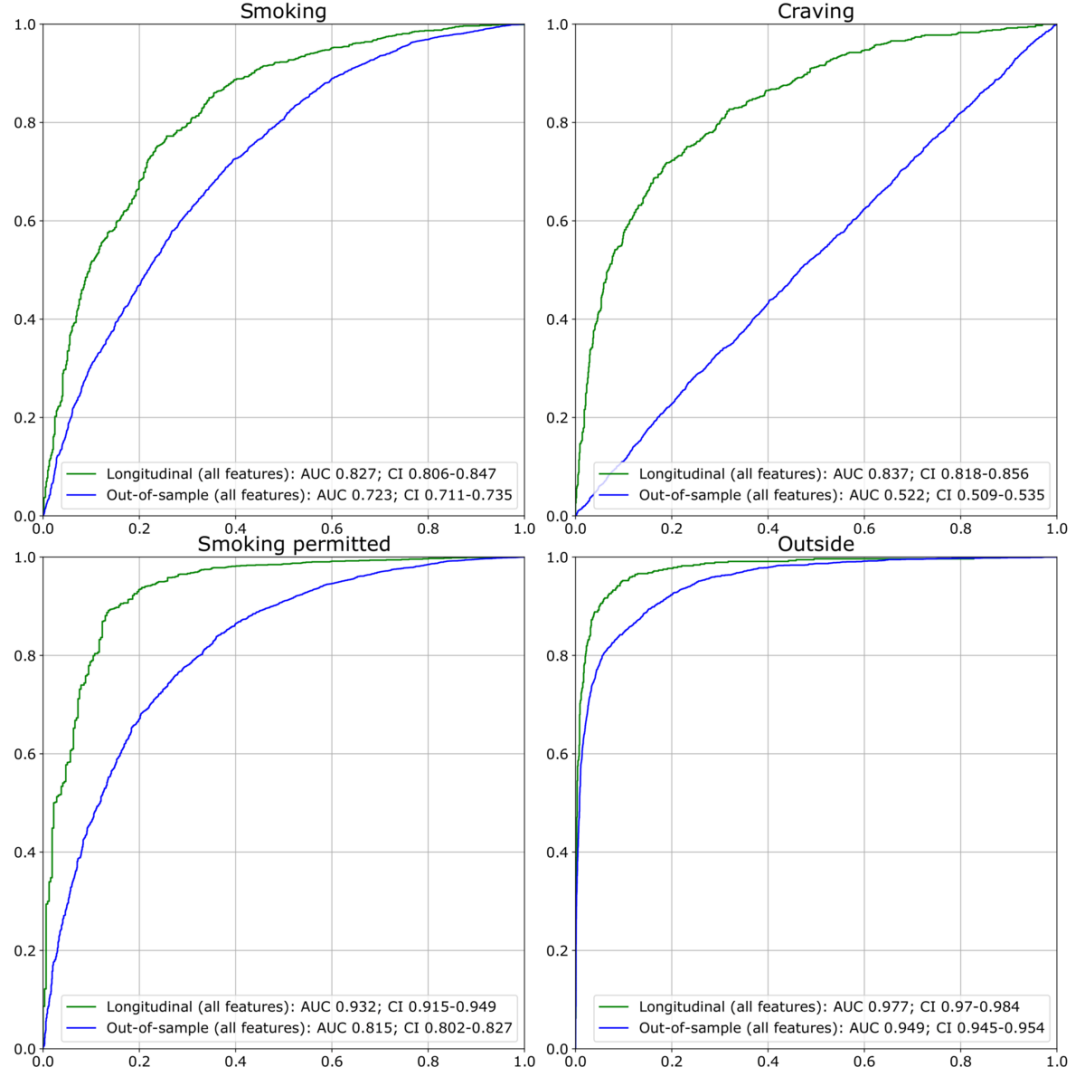
Receiver operating characteristic curves for each of the four outcomes for both the nonpersonalized (out-of-sample) and personalized (longitudinal) models. AUC: area under the receiver operating characteristic curve.

**Figure 3:**
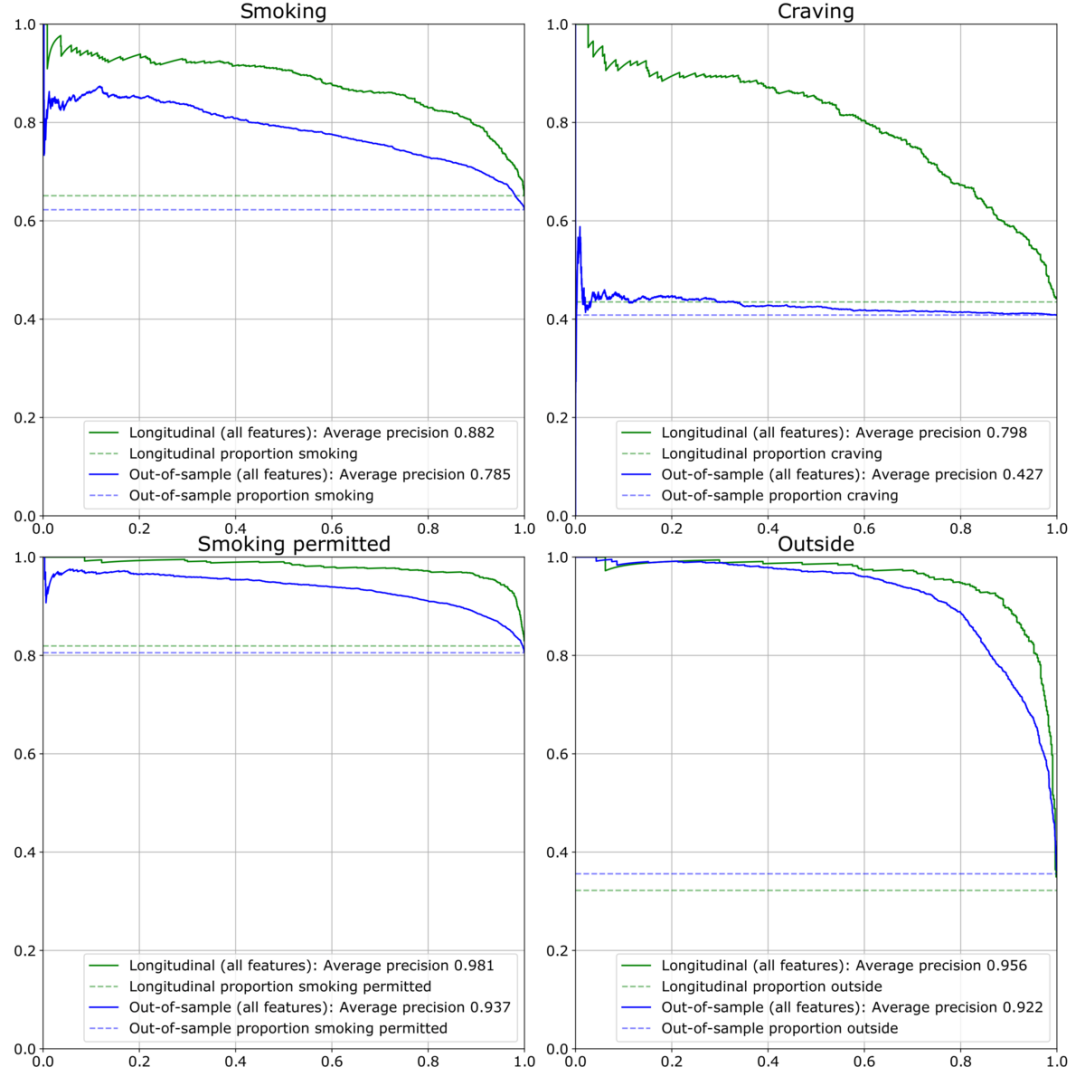
Precision recall curves for each of the four outcomes for both the nonpersonalized (out-of-sample) and personalized (longitudinal) models.

Without personalization (out-of-sample performance), QuitEye predicted smoking with AUC=0.723 and average precision (AP)=0.785, craving with AUC=0.522 and AP=0.427, whether smoking was permitted with AUC=0.815 and AP=0.937, and whether the participant was outside with AUC=0.929 and AP=0.922. With personalization, performance was substantially improved: QuitEye predicted smoking with AUC=0.827 and AP=0.882, craving with AUC=0.837 and AP=0.789, whether smoking was permitted with AUC=0.932 and AP=0.981, and whether the participant was outside with AUC=0.977 and AP=0.956 (Figures 2 and 3).

Image features were critical to these performance figures for all outcomes except craving. In the nonpersonalized (out-of-sample) models, removing the image features lowered AUC by 0.221 when predicting smoking, by 0.229 when predicting whether smoking was permitted, and by 0.253 when predicting whether the participant was outside but by only 0.027 when predicting craving. In the personalized (longitudinal) models, removing the image features lowered AUC by 0.192 when predicting smoking, by 0.168 when predicting whether smoking was permitted, and by 0.178 when predicting whether the participant was outside but increased AUC by 0.034 when predicting craving (Table 2).

In the out-of-sample models, baseline information about household smoking locations improved the prediction of craving (ΔAUC=0.050) and whether smoking was permitted (ΔAUC=0.020), but other nonimage features had less impact. Surprisingly, knowing the time since the last cigarette did not improve the prediction of smoking (ΔAUC=−0.014) or craving (ΔAUC=−0.026; Table 2). Performance among individual participants when predicting smoking was highly correlated with performance predicting whether smoking was permitted (Spearman *ρ*=0.55; *P*<.001), whereas correlations between other pairs of outcomes were not statistically significant.

In the personalized models, the participant identifier substantially improved the prediction of craving (ΔAUC=0.070), and baseline information about smoking locations outside of the household slightly improved the prediction of craving (ΔAUC=0.013); however, nonimage features had little effect on performance (ΔAUC<0.007). Similar to the out-of-sample models, performance among individual participants when predicting smoking was highly correlated with performance predicting whether smoking was permitted (r=0.71; Spearman *ρ*<0.001). However, this was not the case for any other pairs of outcomes.

**Figure 4:**
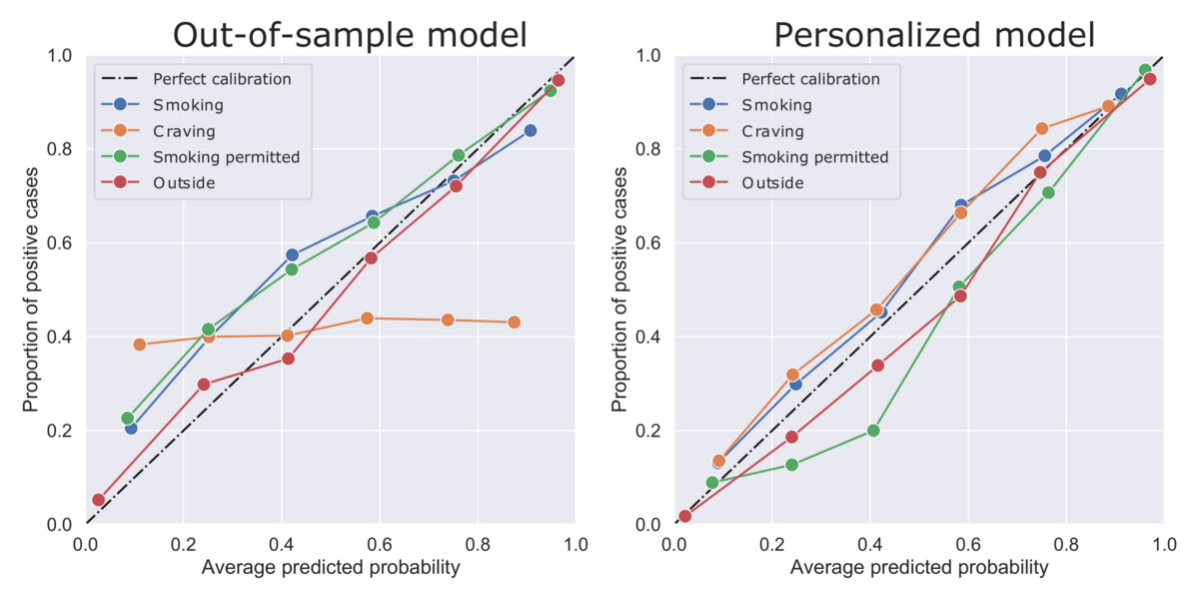
Calibration curves for the nonpersonalized (out-of-sample; left panel) and personalized (longitudinal; right panel) models. Model-predicted probabilities are aggregated by percentile (N=6 bins), then compared with the proportion of positive outcomes in each bin. Good calibration implies that model predictions are an accurate estimate of the true probability of a positive outcome.

Analyses of model calibration showed that outcome probabilities predicted by QuitEye were consistent with true outcome rates, except when predicting craving via the out-of-sample model (Figure 4). This suggests that the predicted smoking probability reflects the true environment-associated smoking probability.

**Table 2:** Model performance (area under the receiver operating characteristic curve) before and after removal of specific data elements.

Model performance			Area under the receiver operating characteristic curve (Δ^a^)						
			Smoking		Craving		Smoking permitted		Outside
**Out-of-sample models**									
	Base model (all features)	0.723 (N/A^b^)		0.522 (N/A)		0.815 (N/A)		0.949 (N/A)	
	Images	0.502 (−0.221)		0.495 (−0.027)		0.586 (−0.229)		0.696 (−0.253)	
	Demographics	0.729 (0.006)		0.542 (0.021)		0.810 (−0.005)		*0.952*^c^ (0.002)	
	Time since last cigarette	*0.737* (0.014)		0.548 (0.026)		*0.819* (0.004)		0.944 (−0.005)	
	Time of day, weekday or weekend	0.726 (0.003)		*0.553* (0.032)		0.806 (−0.008)		0.945 (−0.004)	
	Household smoking locations	0.735 (0.012)		0.472 (−0.050)		0.795 (−0.020)		0.950 (0.001)	
	Other smoking locations	0.717 (−0.006)		0.513 (−0.009)		0.812 (−0.003)		0.948 (−0.001)	
**Longitudinal models**									
	Base model (all features)	0.827 (N/A)		0.837 (N/A)		0.932 (N/A)		*0.977* (N/A)	
	Images	0.635 (−0.192)		0.871 (0.034)		0.764 (−0.168)		0.799 (−0.178)	
	Demographics	0.824 (−0.002)		0.836 (−0.002)		0.929 (−0.003)		0.976 (−0.001)	
	Time since last cigarette	0.828 (0.002)		0.840 (0.003)		*0.938* (0.006)		0.975 (−0.002)	
	Time of day, weekday or weekend	*0.831* (0.004)		*0.844* (0.007)		0.929 (−0.003)		0.975 (−0.002)	
	Household smoking locations	0.826 (0.000)		0.836 (−0.002)		0.925 (−0.007)		0.976 (−0.001)	
	Other smoking locations	0.824 (−0.003)		0.824 (−0.013)		0.929 (−0.003)		0.976 (−0.001)	
	Personal identifier	0.829 (0.002)		0.767 (−0.070)		0.926 (−0.006)		0.975 (−0.002)	

^a^Change in the area under the receiver operating characteristic curve compared to the base model.

^b^N/A: not applicable.

^c^Italics indicate the best performing model for that outcome.

### Effect of Location on Performance

**Figure 5:**
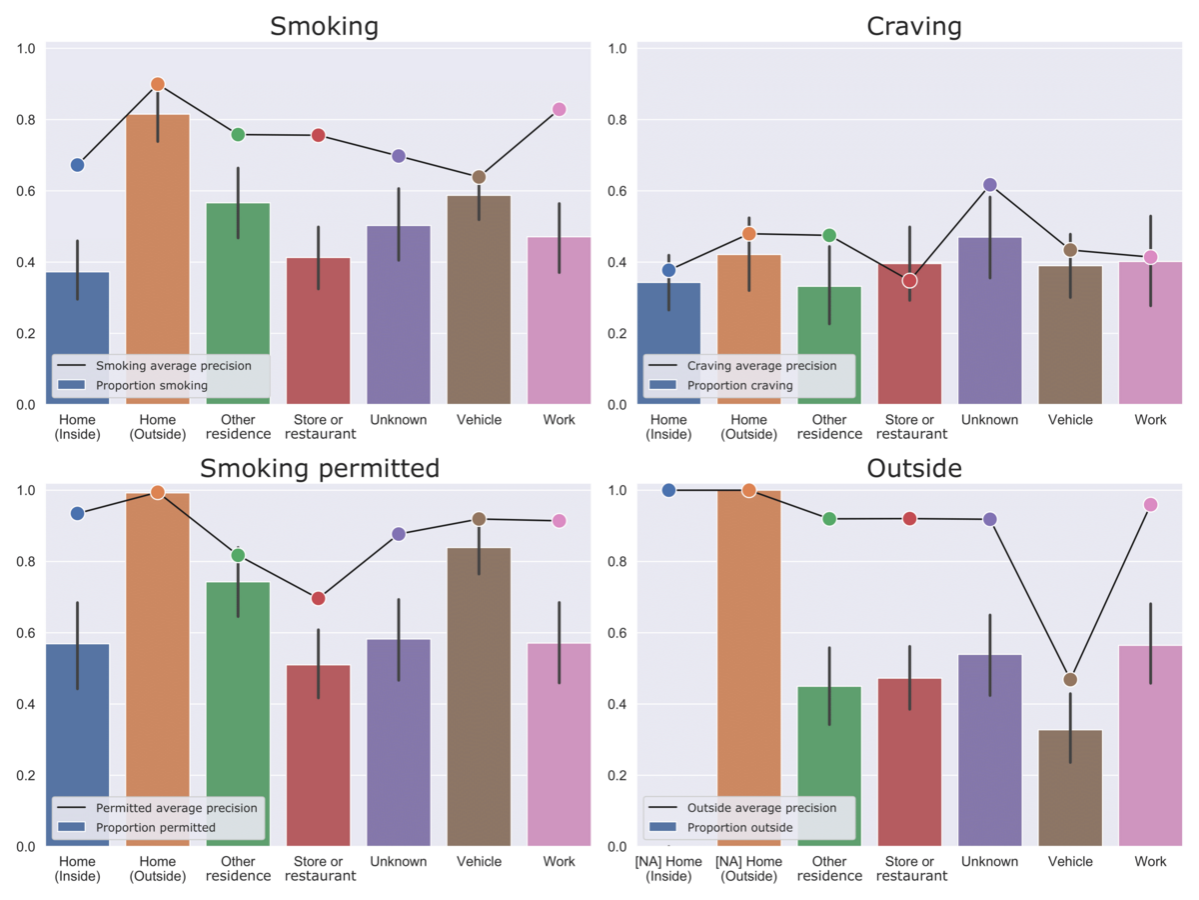
Outcomes and model performance by location type (out-of-sample). The bar plots indicate the proportion of positive outcomes (with SE) by self-reported location type, and the line plots indicate model performance (average precision) for images taken in each location. NA: prediction performance is not applicable, because there is no variability in the outcome in this location type.

Analyses of model performance by self-reported location type showed that QuitEye is more effective in some locations than others. For the nonpersonalized (out-of-sample) model (Figure 5), smoking prediction was most effective at work (AUC=0.848), followed by stores or restaurants (AUC=0.806). Across all prediction tasks, prediction tended to be less effective for images taken within vehicles. Many of these images showed outside scenery as viewed from the vehicle, leading the model to incorrectly predict that the participant was outside (AUC=0.603) rather than inside the vehicle.

**Figure 6:**
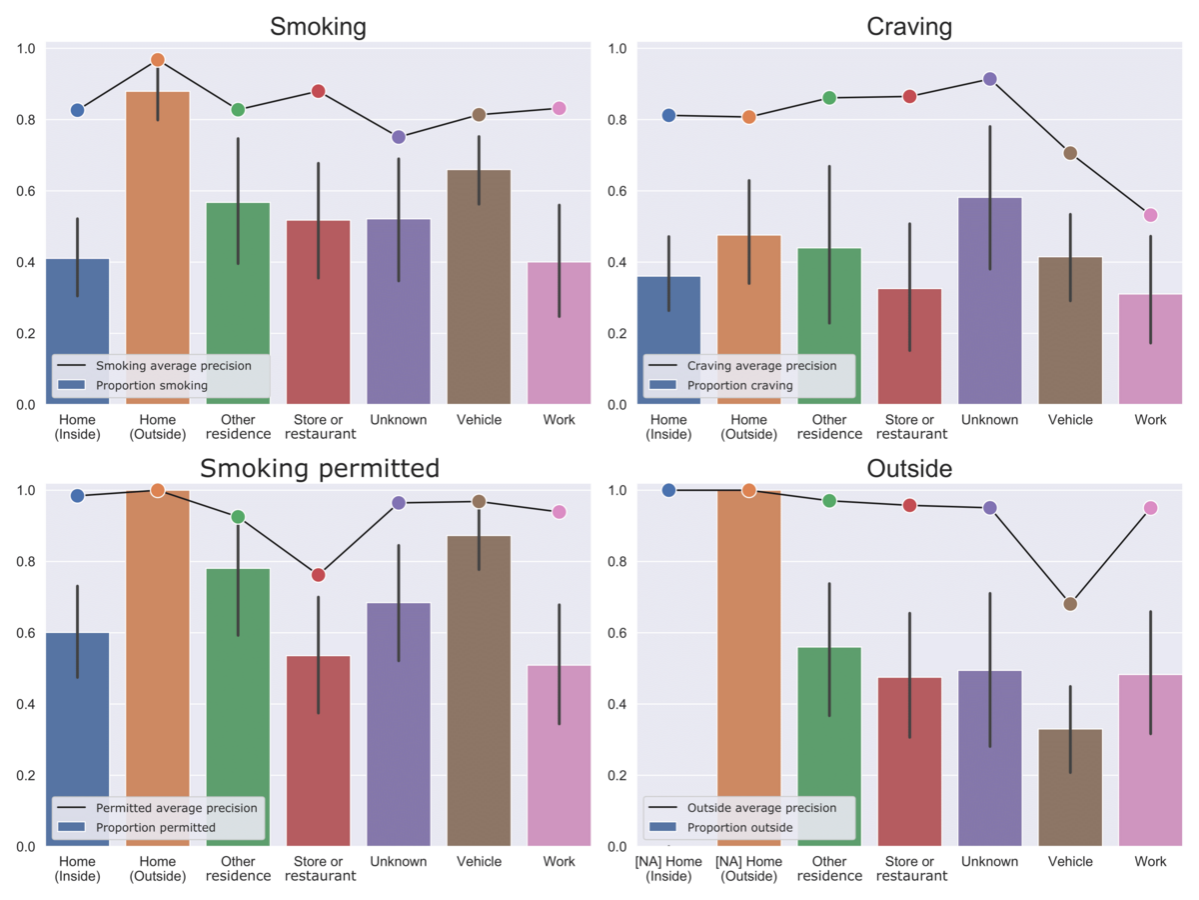
Outcomes and model performance by location type (personalized). The bar plots indicate the proportion of positive outcomes (with SE) by self-reported location type, and the line plots indicate model performance (average precision) for images taken in each location. NA: prediction performance is not applicable, because there is no variability in the outcome in this location type.

Improvements in performance from personalized training also varied by location (Figures 5 and 6). AUC was improved most for outdoor home locations—prediction of smoking was improved by ΔAUC=0.230 in these locations, and prediction of whether smoking was permitted was improved by ΔAUC=0.435.

**Figure 7:**
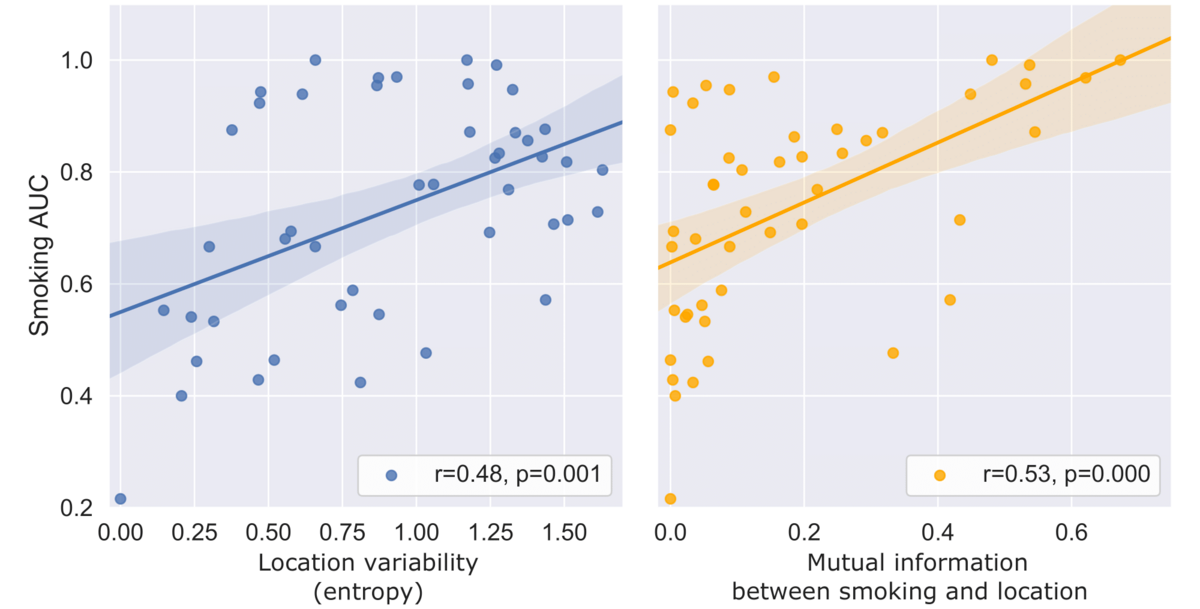
Effect of location variability on performance. Higher performance of smoking risk prediction among individual participants is associated with higher variability in self-reported locations (left panel) and higher mutual information between self-reported location type and smoking status (right panel). AUC: area under the receiver operating characteristic curve.

Smoking prediction was more effective for participants whose self-reported location type was more variable (r=0.48; *P*=.001), quantified as the entropy of self-reported location type. This effect was not observed in other prediction tasks. Smoking prediction was also more effective for those with higher mutual information between smoking and self-reported location type (r=0.53; *P*<.001); the mutual information quantifies the degree to which location type provides information about smoking behavior (Figure 7). This effect was not observed in other prediction tasks.

### Mobile Implementation

**Figure 8:**
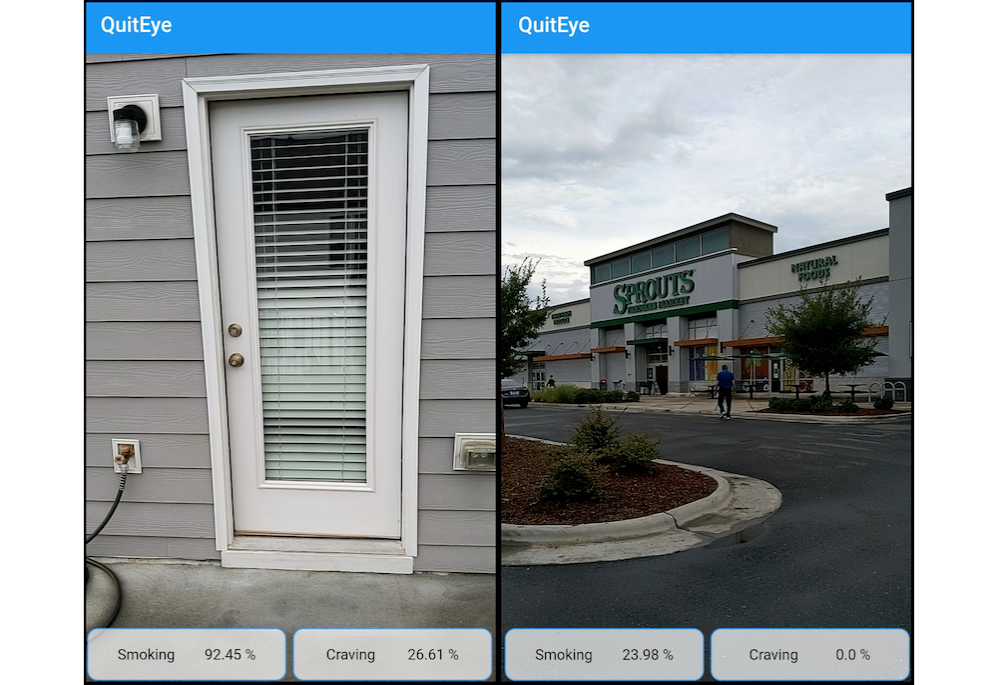
Mobile implementation of QuitEye. Screenshots of real-time smoking and craving risk prediction via the QuitEye mobile app in a high smoking risk environment (left panel) and a low smoking risk environment (right panel).

Screenshots of real-time smoking and craving risk prediction using the QuitEye mobile app are presented in Figure 8.

## Discussion

### Principal Findings

A growing body of knowledge suggests that habitual smoking environments promote craving and smoking behaviors. Our previous study demonstrated that computer vision could distinguish between habitual smoking and nonsmoking environments, leading us to hypothesize that real-world smoking risk has important, quantifiable environmental correlates that can be leveraged to predict smoking behaviors more effectively in real time. This study confirms this hypothesis: QuitEye effectively predicted smoking status and associated outcomes across the full range of environments encountered by our sample of smokers in their daily lives. By learning from other smokers’ behaviors, our models can scan a smoker’s environment to predict their current smoking risk. QuitEye also predicted whether smoking was permitted in the current location and whether the smoker was inside or outside, providing important context relevant to smoking cessation interventions. The results show that knowledge of recent smoking and daily smoking habits (eg, time of day) improved these predictions, but it is the images themselves that contributed most to good prediction performance for all outcomes except craving.

Importantly, these results provide additional evidence that the environmental correlates of smoking vary among smokers. Our nonpersonalized models achieved good out-of-sample prediction performance, suggesting that environmental factors are shared among smokers more than they are distinct. However, model personalization led to statistically significant improvements in smoking risk prediction, implying that there are meaningful environmental correlates of smoking behaviors that are specific to individual smokers.

To achieve personalization in this study, smokers had to self-report their smoking behaviors for 10 days while collecting images of their daily environments. These data were then used to refine the prediction model. This process is burdensome but may be particularly important for smokers whose daily environments are atypical or whose smoking behaviors do not follow common patterns. If this is not possible, a lesser degree of personalization can be achieved by asking smokers to provide information about the locations where they commonly smoke. Alternatively, models can be iteratively improved during use, for example in a mobile app, by prompting the user to confirm or deny smoking predictions made by the model.

The ability to predict environment-associated smoking risk in real time unlocks a range of environment-focused smoking cessation interventions. Real-time risk prediction can be used to trigger a JITAI [9] in which support is provided to smokers via a mobile device at the time and place when it is most needed [31]. Information about environment-associated risk can be combined with information about the smoker’s internal state, provided by wearable devices, to obtain a more comprehensive estimate of smoking risk and craving. We anticipate that information about the external environment would enhance the prediction of smoking risk from other data streams (eg, heart rate from wearable devices), but the degree to which these data sources complement one another has yet to be explored. Ultimately, we envision a system in which wearable eyewear (eg, smart glasses) continuously acquires images of the user’s external environment, alerts them with visual feedback when high-risk environments are detected, and suggests appropriate coping strategies for a given context. As augmented reality technologies mature [22], this paradigm can be used not only to support smoking cessation but also to understand and respond to environmental correlates of a broader range of health-related symptoms and behaviors.

However, several other intervention types are possible. For example, QuitEye could be used to identify environmental correlates of smoking risk in a smoker’s daily environment before a quit attempt, allowing them to restructure their environments or daily activity patterns to increase the likelihood of quitting successfully. As QuitEye can predict the smoking risk associated with any image, including images of locations not yet visited, it can help smokers preemptively avoid visiting prosmoking environments. During a quit attempt, for instance, a smoker might choose to visit a restaurant that has a lower smoking risk, as determined based on images available on the internet.

Although images improved the prediction of smoking substantially, they did not improve the prediction of craving. To the contrary, our best-performing craving prediction models do not incorporate image features, and out-of-sample prediction performance for craving was poor. Our laboratory research suggests that habitual smoking environments do provoke craving, but this study’s results do not provide additional support for this finding. Consequently, the role of environmental factors in the emergence of craving, or in the progression from craving to smoking itself, remains unclear. These conflicting findings may be partly owing to our EMA procedure. At the time of smoking, participants were asked to report their craving *before* smoking. Thus, the corresponding image taken at the time of smoking may not match the external environment corresponding to self-reported craving. However, in a follow-up analysis, we attempted to predict craving only from random prompts, and similar AUC values were observed. Further studies are needed to examine the environmental correlates of craving more thoroughly. In particular, continuous image acquisition (via wearable cameras or smart glasses) may provide additional environmental or social cues that are relevant to craving but not captured by a single image. Alternatively, general craving may be driven more by internal (eg, low plasma nicotine levels and negative affect) rather than external factors.

As shown in Figure 7, smoking risk prediction was more effective for participants whose locations were more variable and for whom location type provided more information about smoking behavior. Some participants’ smoking environments were mostly distinct from their other daily environments, allowing our models to identify a strong relationship between environmental factors and smoking status. Other participants tended to smoke in the same environments where they spent most of their time, making it difficult to identify robust environment-smoking associations. Notably, this difference was not associated with demographic factors in this study. More studies are needed to understand these groups and determine whether there are identifiable subpopulations of smokers among whom QuitEye is particularly effective or ineffective.

### Limitations

Although EMA provides more accurate smoking tracking than other self-report methods [32], smoking events may have been omitted or incorrectly reported. Our models predicted self-reported smoking, which may differ from true smoking events. Although our EMA procedure was designed to be brief and minimally burdensome, picture-taking and other EMA requirements may have increased the number of smoking events omitted by our participants. Furthermore, some EMA responses may not have been completed promptly upon smoking, thus reducing self-report validity. Other outcomes were also self-reported and subject to participant error or omission. As previously discussed, our EMA asked participants to self-report their craving *before* smoking in the smoking-initiated prompts, which may have introduced additional errors or variability.

In addition to smoking-initiated prompts, participants completed a total of six system-initiated prompts at randomly selected times throughout the day. More frequent prompts would have provided a more comprehensive sample of participants’ daily environments, but this might have also resulted in reduced EMA adherence. Camera design and image quality varied among participants, who used their own smartphones to take pictures. Variability in image quality can be reduced by acquiring images using wearable cameras or smart glasses. This approach would also allow images to be captured throughout the day, providing complete information about the participants’ daily environments.

### Future Directions

This study did not include a quit attempt. The results showed that QuitEye predicts smoking risk effectively outside of a quit attempt, but its ability to predict lapses and relapse after quitting is unknown. Other (nonimage) data streams from mobile devices have been used to predict lapse risk [21], and we anticipate that QuitEye would provide complementary information about environment-associated risk factors. In future work, we will explore the relationship between the environmental correlates of smoking before quitting and the environmental correlates of lapse and relapse.

Owing to privacy concerns, participants were asked to avoid taking pictures of other people. However, this restriction may have prevented us from identifying interpersonal triggers and other important social determinants of smoking. The ability to recognize these and other dynamic environmental features is an important advantage of our approach compared with other sources of environmental information, such as GPS. In future work, we hope to explore the use of computer vision to identify the social determinants of smoking.

Now that QuitEye has been implemented as a mobile app, we can prospectively evaluate the real-time prediction of environment-associated risk and develop *environment-aware* mobile health cessation interventions. These interventions will incorporate information about the environment in addition to user physiology and other information with recognized predictive values [11-15]. An important goal of this study is to quantify the contribution of each data source (eg, physiology vs environment) to overall prediction performance. Initial interventions incorporating QuitEye will use smartphone cameras to identify environment-associated smoking risks in smokers’ daily environments. Later interventions will use smart eyewear to continuously acquire images of the smoker’s environment and provide support when high-risk environments are encountered. An important component of this study will be to evaluate the benefit of just-in-time support versus the cost of false alarms, which will allow us to select an appropriate risk threshold for triggering the intervention.

### Conclusions

Images of daily environments can be used to predict smoking risk effectively. Our risk prediction system, QuitEye, also predicts craving, whether smoking is permitted, and whether the participant is outside, providing important contextual information that could inform JITAIs for smoking cessation. Performance can be further improved through personalization, achieved by (1) fine-tuning QuitEye with images of a given smoker’s daily environment or (2) asking participants to provide information about their habitual smoking environments. QuitEye has been optimized for mobile devices and implemented as a mobile app, allowing environment-associated smoking risk to be continuously assessed in a mobile device-based smoking cessation intervention.

## Abbreviations

AP: average precision
AUC: area under the receiver operating characteristic curve
EMA: ecological momentary assessment
JITAI: just-in-time adaptive intervention
photoEMA: photograph-augmented ecological momentary assessment
